# Clinical Lecture

**Published:** 1892-04

**Authors:** G. Frank Lydston

**Affiliations:** Chicago; Professor of Genito Urinary Diseases and Syphilology in the College of Physicians and Surgeons


### CLINICAL LECTURE.

BY G. FRANK LYDSTON, M. D., CHICAGO.

Professor of Genito-Urinary Diseases and Syphilology in the College of Physicians and Surgeons.

### URTICARIA COMPLICATING SYPHILIS.

*Gentlemen*—The next case to which I will call your attention this morning, is one which illustrates in a very graphic manner the complexity of some of the problems in diagnosis which occasionally confront the dermatologist. This patient, who is about twenty-five years of age, is as you see very badly nourished, and for excellent reasons. He has been a member of a small theatrical organization which, as he graphically expresses it, "busted a few weeks ago." By industrious counting of railroad ties he managed to reach Chicago, and since he arrived here has eked out a precarious livelihood by doing small jobs of work, and at night has been sleeping in a barn with several other unfortunate histrionic wrecks. His clothing has been scanty, and he has suffered from the extremely cold weather which we have been having, as well as from lack of proper food. He states that some three months ago he had a chancre, followed in a few weeks by a sore throat and scabs in his scalp with falling of the hair. He states that he did not notice until a few weeks ago any eruption upon his skin. He has several small pimples upon his shoulders, back and breast which he has had ever since he can remember. He has been treated with mercury and iodide of potassium according to his statement since the onset of what was probably syphilis.

About three weeks ago the patient began to be troubled by intense itching of the skin, unattended at that time, he says, by any eruption, which itching was greatly aggravated at night. This has continued until the present time, and within a few days has been greatly increased in severity coincidentally with the appearance of large blotches most marked upon his limbs.

We will now have him remove his clothing and investigate the character of this eruption. You will notice as the most conspicuous dermic lesion, an abundance of crusts formed by dry blood here and there which are quite characteristic of nail scratches. Upon the arms, forearms and thighs will be observed a number of pink and white elevations of irregular form which, he says, itch and burn constantly. On inquiry we find that these lesions appear and disappear in various locations within a very short time. For example, he states that early this morning there were no blotches upon his forearms. The merest tyro would scarcely fail to recognize the character of these lesions. They constitute the typical eruption of urticaria. I notice here and there a faint mottling or pigmentation of the skin which is suspicious of a previous syphilitic lesion. I will now ask the patient to step away to the rear of the amphitheatre and would like you to look steadily at the polymorphous eruption upon his skin. As the patient recedes from you, you will observe that the nail marks and urticarial eruption gradually fade. The same is true of the eruption of papules interspersed with a few pustules to which I will call your attention, upon the shoulders, back and breast. The faint pigmentation of the skin, on the contrary, becomes more prominent as the patient recedes, and we see plainly an eruption beneath the skin of the characteristic ham-red color approximating here and there a coppery hue characteristic of fading roseola. The inguinal, epitrochleal, and cervical glands are characteristically indurated and enlarged, and on examination of the mouth and throat we find the peculiar congestion of the fauces so familiar in secondary syphilis, and several mucous patches upon the tongue. The hair, you will observe, has fallen out in marked patches here and there (alopecia areata). We have here then a complex eruption. In the first place, we have the lesions produced by scratching for the purpose of relieving the intense general pruritis. Secondly, the lesions of acne vulgaris. Thirdly, those of ordinary urticaria. Fourthly, a fading roseola syphilitica.

I wish to call your attention to the practicality of the method which I have adopted in this case of subtracting, so to speak, the element of confusion afforded by the non-specific lesions from the syphilitic eruption *per se*. The manner in which the syphilitic eruption became more prominent and the non-specific eruptions less prominent as the patient receded from you should certainly be an excellent object lesson. The original difficulty in this man was undoubtedly syphilis. As a consequence of privation and exposure to inclement weather, a certain degree of nervous disturbance of the skin has resulted with the production of that form of pruritis which, for want of a better term and on account of its coexistence with the onset of cold weather, we have termed winter itch. It is probable that this irritation of the skin associated with that produced by vigorous scratching has developed the urticaria; there may possibly be some gastro-intestinal disturbance as an additional factor.

The line of treatment which should be followed in this case is more simple than practicable. The patient should be well housed, fed, and warmly clothed, and should take frequent warm baths containing starch and carbonate of soda in solution. The only remedies suitable for internal medication in this case at the present time are tonics of various kinds. The case is important chiefly from a diagnostic standpoint and shows how exceedingly careful we must be in dermatological work.

### CIRCUMCISION IN A CASE IN WHICH THE OPERATION HAD BEEN PREVIOUSLY IMPROPERLY PERFORMED.

The next patient presents a very interesting condition as showing how a supposedly simple operation can be bungled by a careless and incompetent operator. This boy of twelve years of age has just passed through the hands of a firm of advertising quacks in this city who attempted to perform circumcision upon him. The mother states that he is worse than ever, and has therefore brought him to the clinic this morning to ascertain why he is worse than before the operation, and cannot pass his water as readily as before. Upon inspection of the affected organ you will observe that the prepuce is very tightly drawn forward over the glans and that I cannot retract it even by using considerable force. The preputial orifice presents evidences of the operation which the boy has undergone in the form of a puckered circular cicatrix through the lumen of which I can barely introduce a probe. The cause of this condition is evident. The person who performed the operation simply drew the foreskin forward and snipped it off, after which he allowed the part to heal at its own sweet will. The only thing to do is to rectify the damage done by the previous attendant. We will proceed by splitting up the prepuce upon the dorsum, trimming off the scar tissue, turning back the flaps of mucous membrane and stitching them to the penile integument in the same manner as in a primary circumcision. It is frequently recommended by excellent authorities to omit stitches in the circumcision of children; but I would advise you to always insert them unless some special contra-indication exists. In performing this operation be sure and allow plenty of room for subsequent expansion of this organ. This may be done by making a short longitudinal incision in the proximal flap, *i. e.* in the penile portion of the skin; the corners of skin which are left should be trimmed off with the scissors.

